# Social distancing slows down steady dynamics in pedestrian flows

**DOI:** 10.1063/5.0062331

**Published:** 2021-10-19

**Authors:** Kelby B. Kramer, Gerald J. Wang

**Affiliations:** Department of Civil and Environmental Engineering, Carnegie Mellon University, 5000 Forbes Avenue, Pittsburgh, Pennsylvania 15213, USA

## Abstract

Amidst the ongoing pandemic, social distancing has been broadly adopted as an effective front-line defense strategy for mitigating disease transmission. Viewed through the lens of particle-based simulations of flow, the practice of social distancing corresponds to a (significant) increase in an internal length scale of the flow, namely, the radius within which particles (pedestrians) strongly repel fellow particles. In this study, we report the results of two-dimensional pedestrian dynamics simulations modeling pedestrian counter-flows under confinement, in which individual pedestrians are described as active particles that aim to maintain a target speed while avoiding collisions. By systematically varying two quantities—the pedestrian density and the degree of social distancing—we compute fundamental diagrams for confined and socially distanced pedestrian flows, which show average pedestrian speed as a function of density and social distancing. These results reveal the sensitive dependence of average velocity on both independent variables, including a social distancing-induced jamming transition. These results highlight the need for both deliberate planning and careful public-health messaging regarding social distancing as shared indoor spaces return to appreciable levels of occupation.

## I. INTRODUCTION

Flows of pedestrians are intricate phenomena governed by complex dynamics at a variety of length scales, ranging from interpersonal interactions that play out on the scale of tens of centimeters^1,2^ all the way out to long-wavelength patterns that manifest on the order of tens of meters.^3^ The goal of the present study, motivated by the ongoing COVID-19 pandemic, is to study the relationship between social distancing and the dynamics of pedestrian flows in corridors by studying the effect of two critical length scales that affect flow: One length scale characterizing individual desires for personal space and another for the confining environment. The central question of this work is: How does strong adherence to social distancing affect large-scale pedestrian counter-flows (two groups of pedestrians walking in opposite directions in a shared space)? In particular, we investigate the effects of increasing an internal length scale of a pedestrian flow (namely, the degree of personal space maintained by each pedestrian); one might intuitively expect interesting phenomena to emerge as that length scale begins to approach the characteristic length scale of confinement.

This work builds upon a significant body of work in recent years that has studied the effect of a variety of factors in the context of pedestrian counter-flows, including obstacle arrays,^4,5^ spontaneous lane formation,^6^ internal ordering,^7,8^ and psychological groupings.^9^ A topic of particular interest in pedestrian dynamics studies is the characterization of jamming or clogging phenomena in relatively narrow corridors,^7,10–12^ which we study here.

For the sake of clarity, it is worth noting that we do not comment in this work on the efficacy of various social distancing guidelines for reducing disease transmission, a topic that is vastly beyond the scope of this work. We are particularly keen to emphasize that there is significant evidence that the “six-feet rule” alone is not sufficient to prevent (airborne and, in some cases, even droplet-borne) transmission of SARS-CoV-2 (see, e.g., work on airborne transmission^13–15^ and droplet-borne transmission^16,17^), which can be further complicated in the confined environments we study in this work by air conditioning and ventilation.^18,19^ Nevertheless, because of consistent public-health guidance^20^ that features six feet (or, in metric-system societies, 1.5 or 2 m) as the recommended degree of social distancing, there is a specific distancing length scale in the broad public consciousness. Our focus here is on changes in the dynamics of a pedestrian flow when an internal length scale of that flow (one that ultimately originates from pedestrian psychology and not from detailed knowledge of epidemiology or environmental fluid mechanics) is significantly increased.

## II. METHODOLOGY

### A. Governing equation

**FIG. 1.**
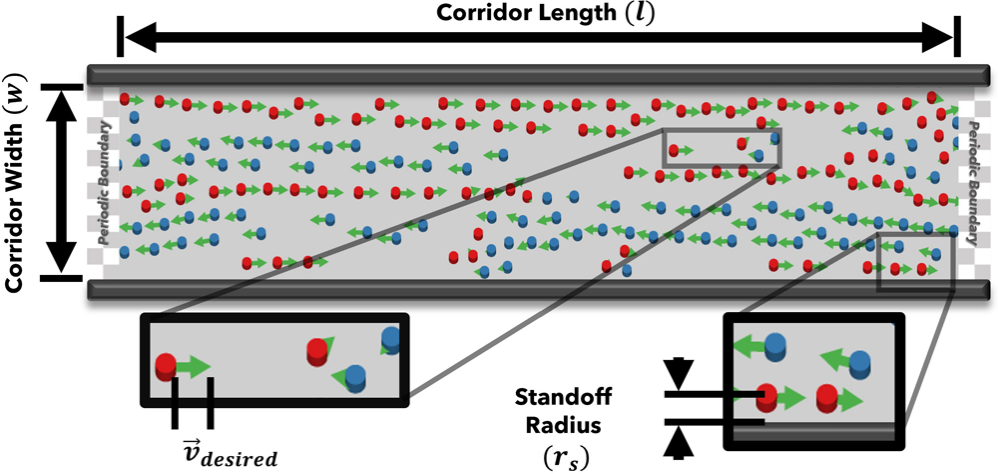
Pedestrians flowing to the left (blue) and right (red) in a corridor of width *w*. At the start of each simulation, all left-flowing pedestrians are placed to the right of the corridor centerline (and vice versa). Periodic boundary conditions are applied at each end of the hallway. Arrows (green) indicate each pedestrian's instantaneous velocity, which (approximately) equals ${\vec{v}}_{\text{desired}}$ for pedestrians with no nearby neighbors (inset to the left). Due to a repulsive pedestrian-wall interaction, pedestrians maintain a minimum characteristic standoff radius *r_s_* from the corridor walls (inset to the right). Note that this (steady) flow exhibits clearly separated lanes, which spontaneously form after a period of transient dynamics in systems at sufficiently low pedestrian density.

In the present study, we use the Large-scale Atomic/Molecular Massively Parallel Simulator (LAMMPS)^21^ to perform two-dimensional (2D) particle-based simulations of confined pedestrian counter-flows in corridors of fixed width, as schematically illustrated in Fig. 1.

In each simulation, two groups of pedestrians (*N* in total) move in opposing directions (both groups are of equal size, and all pedestrians are identical except for their intended direction of flow). We use a social-force model^1,22^ to describe inter-pedestrian interactions and pedestrian-wall interactions. Social-force-based models have been demonstrated to be in reasonable agreement with laboratory and field studies (see, e.g., Zhang *et al.*,^8^ Seer *et al.*,^23^ and Sticco *et al.*^24^). In particular, the force on each pedestrian is given by

$$\begin{array}{c} \vec{F}=m\frac{{\vec{v}}_{\text{desired}}-{\vec{v}}_{\text{inst}}}{\tau }-\vec{\nabla }U_{\text{SD}}+{\vec{F}}_{\text{near}}+\vec{R}. \end{array}$$ (1)

The first term serves as a proportional controller, which generates a force to reduce deviations between each pedestrian's desired velocity ${\vec{v}}_{\text{desired}}$ and their instantaneous velocity ${\vec{v}}_{\text{inst}}$, with adjustments happening over a characteristic relaxation timescale *τ*. The second term features the inter-pedestrian energy, which (critical to the present study) encodes pedestrians' desired degree of social distancing. The third term represents near-field interactions between pedestrians in physical contact with one another. The fourth term constitutes random noise (zero mean and delta correlated), which serves to prevent inter-pedestrian deadlocking.

More details on our pedestrian dynamics simulations are provided in the Appendix; here, we summarize in brief the most physically relevant quantities for contextualizing the results presented below. In all of our simulations, we set the ratio m/τ to 1.05 kg/s; $\left||{\vec{v}}_{\text{desired}}|\right|$ to 1.5 m/s; parameters for ${\vec{F}}_{\text{near}}$ to be identical to those used by Helbing *et al.*,^25^ and the variance of $\vec{R}$ to be 2 square-Newtons. It is worth noting that in our systems, because pedestrians are imbued with a significant preference for social distancing, our dynamics are not expected to (and have been checked to be insensitive to) the exact values of the parameters in ${\vec{F}}_{\text{near}}$.

### B. Independent variables

Across the simulations performed in this work, we vary three quantities, each of which we describe in detail below: the inter-pedestrian potential (social interaction energy), the pedestrian density, and the corridor width.

The social interaction energy $U_{\text{SD}}$ is implemented as an exponential potential with an attenuation length scale *d*_0_ (this interaction is similar in spirit to the Buckingham potential^26^ used in molecular simulation). In particular, the inter-pedestrian energy for pedestrian *i* is given by

$$U_{\text{SD}}^{\left(i\right)}(r_{ij})=\sum_{j\neq i}Ke^{-\frac{r_{ij}}{d_{0}}},$$ (2)

where *r_ij_* is the separation distance between pedestrians *i* and *j*. Throughout this work, *K* = 2000 J; *d*_0_ is varied in the range 0.1 m $\leq d_{0}\leq 0.2$ m. This range of values was chosen so that pedestrians experience an appreciable social-distancing force (defined as a force sufficient to produce an acceleration exceeding $10^{-2}\;g$) at distances ranging from 1 ( $d_{0}=0.1$ m) to 2 m ( $d_{0}=0.2$ m); this range is motivated by typical pre-pandemic levels of social distance^2^ and public-health guidance issued by the World Health Organization (WHO) and Centers for Disease Control and Prevention (CDC).^20^

We also vary the density of pedestrians within the corridor. Throughout Sec. III, we report an excluded-area-corrected density,

$$\phi_{\text{exc}}=\frac{N}{A-N\pi d_{0}^{2}-2r_{s}l},$$ (3)

where *A* = *lw* is the hallway area, *l* = 40 m, $r_{s}=0.3$ m is the characteristic standoff distance between pedestrians and the confining walls, and, as a reminder, *d*_0_ is the social distancing length scale. This expression for density reduces the system area by the total area that is inaccessible to pedestrians, owing to the steep increase in repulsive interactions as pedestrians approach a separation distance of *d*_0_ (in the spirit of, e.g., the van der Waals equation of state, which adjusts the volume of a system by the amount of space rendered inaccessible due to molecules having finite size). This method for reporting density naturally captures dynamical similarities between increasing the total number of pedestrians and increasing each pedestrian's desire for social distancing (both lead to an increased sensation of “crowding”). Our simulations operate in the range $0\leq \phi_{\text{exc}}\leq 2$ m^−2^. Simulations were performed in a range of corridor widths, with w∈{3.5,5.5,7.5,12.5} m.

### C. Simulation output

Each simulation is run for at least 20 s (using a velocity-Verlet integrator with a time step of $dt=3\times 10^{-3}$ s) to achieve steady pedestrian flow, after which kinematics are recorded for 30 s. In each simulation, the key quantity of interest is the average pedestrian speed; averages are computed over both populations of pedestrians (those transiting to the left and those transiting to the right).

In order to quantify uncertainties associated with statistical sampling, for each combination of $\left\{d_{0},\phi_{\text{exc}},w\right\}$, 512 simulations were performed, each differing in initial pedestrian positions. Including variations over 11 values of *d*_0_, 20 values of $\phi_{\text{exc}}$, and four values of *w*, we performed a total of 450 560 simulations. Trajectories were visualized using OVITO.^27^

## III. RESULTS AND DISCUSSION

**FIG. 2.**
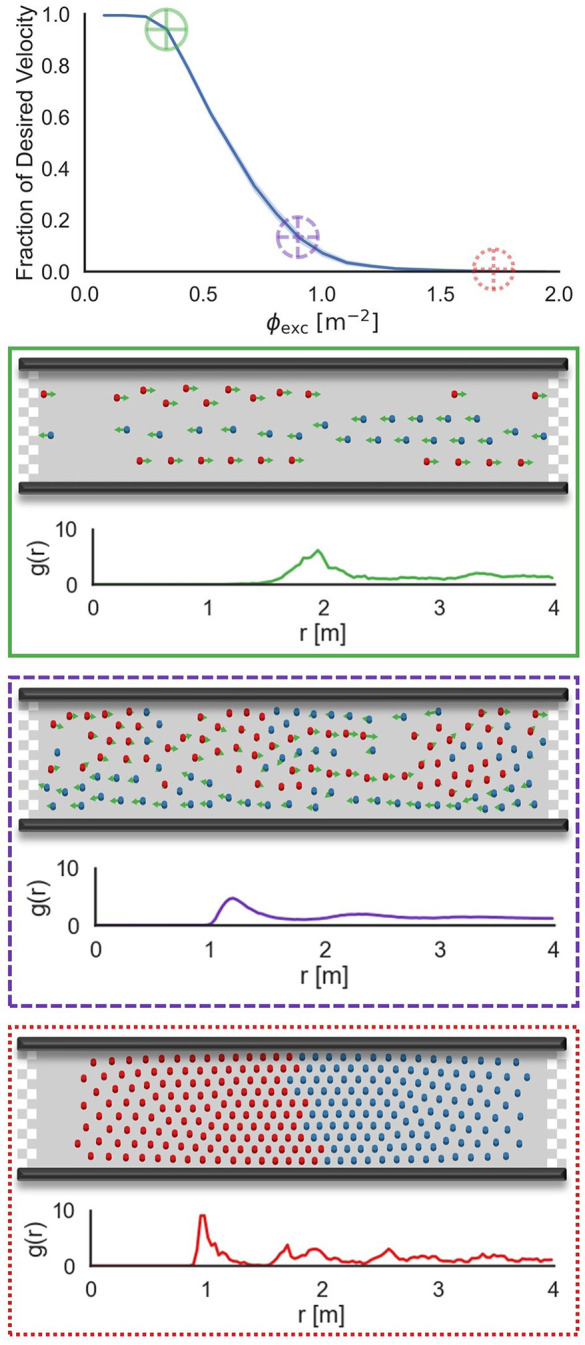
Fundamental diagram showing average pedestrian speed (normalized against $\left||{\vec{v}}_{\text{desired}}|\right|$) as a function of $\phi_{\text{exc}}$ for $d_{0}=0.15$ m and *w* = 5.5 m. For three specific values of $\phi_{\text{exc}}$, snapshots from the pedestrian flow are shown, along with the inter-pedestrian radial distribution function *g*(*r*). From top to bottom, the three conditions of pedestrian density illustrate: unimpeded flow (green); significantly hindered flow near the onset of jamming (purple); and a fully jammed system (red).

Our first set of results is presented in the form of a fundamental diagram, which shows average pedestrian speed as a function of pedestrian density (see, e.g., Ref. 7 as a helpful reference for background on fundamental diagrams). We begin in Fig. 2 by presenting a single fundamental diagram for a social distance length scale of $d_{0}=0.15$ m and corridor width of *w* = 5.5 m, which motivates several observations:

(1) At relatively low densities (in the case of Fig. 2, $\phi_{\text{exc}}≲0.4$ m^−2^), pedestrian “near-collisions” (more specifically, pedestrian interaction events that cause non-negligible deviations in trajectory) are rare and pedestrians are easily able to (spontaneously) form lanes, within which they can maintain their target speeds. The peak in the radial distribution function near 2 m indicates that typical inter-pedestrian separations are substantially higher than the separation distance at which $U_{\text{SD}}$ becomes appreciable [to provide a sense of scale, at a separation of 2 m, two pedestrians each experience a negligible acceleration that is $O(10^{-4})g$]. As such, this case is representative of the dilute (noninteracting) limit.
(2) At intermediate densities (in the case of Fig. 2, $0.4≲\phi_{\text{exc}}≲1.0$ m^−2^), inter-pedestrian interactions are no longer negligible. Near-collisions are commonplace and pedestrians spend appreciable amounts of time maneuvering laterally to maintain social distance. As a consequence, the (normalized) average pedestrian speed falls significantly below unity. Interestingly, the radial distribution function in these cases is reminiscent of a liquid,^28^ featuring a single pronounced “coordination shell” and a low degree of ordering outside this shell. By balancing the two most significant forces from Eq. (1),
$$\left\|m\frac{{\vec{v}}_{\text{desired}}-{\vec{v}}_{\text{inst}}}{\tau }\|=\|\vec{\nabla }U_{\text{SD}}\right\|,$$
  for a pair of pedestrians, we predict that the location of the first coordination shell should occur just inside of
$$r_{\text{peak}}=d_{0}\;\text{ln}\;\frac{K\tau }{d_{0}m||{\vec{v}}_{\text{desired}}||},$$
  an upper bound since $||{\vec{v}}_{\text{inst}}||>0$ at intermediate densities. This prediction is supported by our simulation results: For the parameters corresponding to Fig. 2, $r_{\text{peak}}=1.4$ m.
(3) At high densities (in the case of Fig. 2, $\phi_{\text{exc}}≳1.0$ m^−2^), the system passes through a jamming transition. The pedestrian counterflow is arrested and lanes are never able to develop as none of the external forces on the system are of sufficient magnitude to overcome the force associated with one pedestrian penetrating into the counterflowing phase (a small subset of simulations was run for three orders of magnitude longer to verify this claim of arrested dynamics). Furthering the condensed-matter analogy from above, at such densities, the system exhibits structure and dynamics similar to a solid; in particular, the radial distribution function exhibits several discernible peaks. Although this specific analogy with the solid phase is not the focus of our manuscript, we nevertheless note that the highest-density system of pedestrians shown in Fig. 2 would meet the Hansen-Verlet criterion for freezing.^29^ The analogy with crystallization (including the emergence of topological defects, as can be observed at the bottom of Fig. 2) was studied in beautiful work recently by Cheng *et al.*^30^

**FIG. 3.**
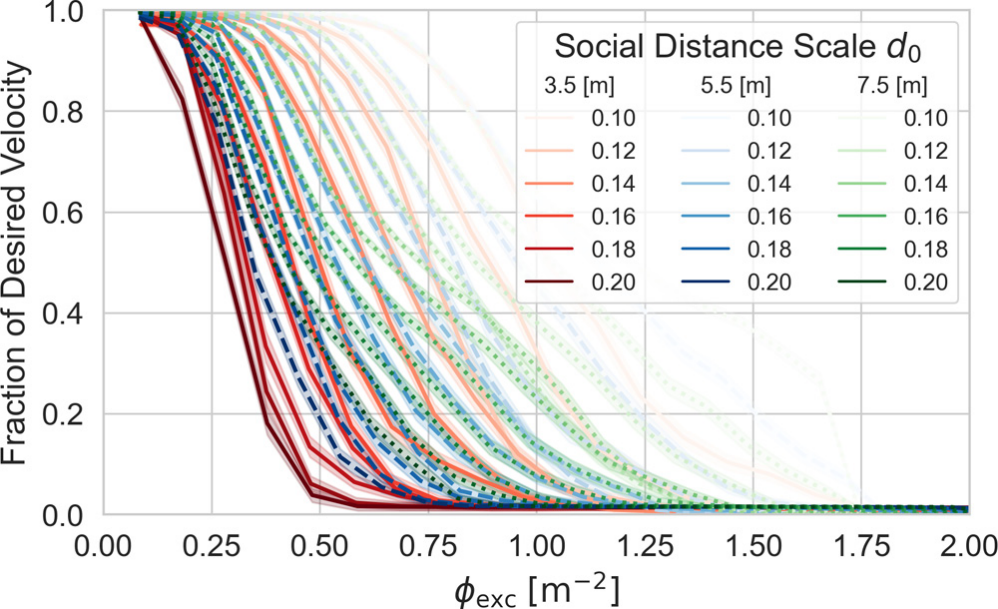
Three families of fundamental diagrams, each containing 11 individual diagrams for differing values of *d*_0_. Line color and style identify the value of *w* as 3.5 m (red/solid), 5.5 m (blue/dashed), or 7.5 m (green/dotted). Shade indicates the value of $d_{0}$, with darker shading corresponding to a larger social distancing length scale. For each fundamental diagram, the 95% confidence interval (based upon 512 statistically independent simulations differing in initial pedestrian positions) is also shown.

In Fig. 3, we show several families of fundamental diagrams; each line represents an individual fundamental diagram for a particular choice of corridor width and social distancing length scale. Based upon these data, we make several remarks:

1. The pedestrian density at the onset of jamming decreases with increasing degree of social distancing. Furthermore, narrow corridors promote jamming: Although this is an unsurprising observation, it is intriguing that merely correcting for the area excluded by pedestrians' “hard” social distancing core (of area $\pi d_{0}^{2}$) does not scale out the dependence of jamming on the corridor width.
2. This phenomenon is reminiscent of certain jams in microfluidic^31^ and granular^32^ systems. We note that this clogging is exclusively due to “steric” interactions, as there are no attractive inter-pedestrian interactions within our model. It is also worth noting that the transition to jamming for our pedestrian flows (governed by a social-force model with an exponentially decaying energy) does not occur at a universal threshold (for comparison, the onset of jamming in randomly close-packed hard disks in 2D is at an occupied area fraction of 0.82^33^ and is within 10% of this value for a variety of soft systems^34^). The detailed analysis by Chraibi *et al.*^11^ highlights the subtlety of this phenomenon, even in the simpler case of pedestrian flows in one dimension.
3. The sharpness with which the jamming transition occurs depends strongly on social distancing. In particular, for the smallest values of *d*_0_ studied, substantial decreases in average pedestrian speed only occur if $\phi_{\text{exc}}$ increases by O(1) m^−2^; for the largest values of *d*_0_ (which, as a reminder, correspond to pedestrians first experiencing an appreciable social force at a separation distance of 2 m), substantial decreases can occur with changes in $\phi_{\text{exc}}$ that are O(0.1) m^−2^. This result underscores the sensitivity of pedestrian congestion to even modest changes in mutually shared preferences for social distancing.

**FIG. 4.**
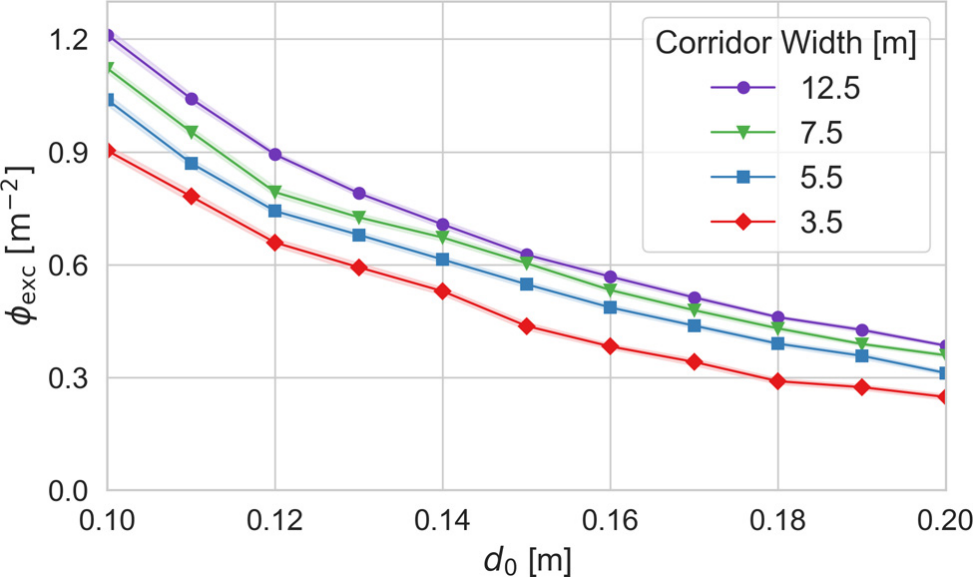
Pedestrian density at which average speed falls to half of target speed, as a function of $d_{0}$, for four corridor widths. For each curve, the 95% confidence interval (based upon 512 statistically independent simulations) is also shown.

Building upon this last remark, in Fig. 4, we identify the density at which average pedestrian speed first falls to half of the target speed, as a function of the social distancing length scale. These plots highlight the nonlinear dependence of the jamming threshold on the degree of social distancing: In the narrowest corridor, a mere 10% increase in the social distancing length scale can shift the onset of jamming by nearly 20%. At low levels of social distancing and in wide corridors, $\phi_{\text{exc}}$ can reach 1 m^–2^ before the flow crosses this jamming threshold; for $d_{0}=0.2$ m, this threshold is reached at densities below 0.5 m^–2^. In the language of transportation engineering, these thresholds correspond to levels of service of B or C, and a level of service of D,^35^ respectively.

Taken collectively, these results point to the strong sensitivity of pedestrian flow dynamics to even small increases in the characteristic amount of social distancing sought by each pedestrian. These results also indicate that as a consequence of steric effects during flow, in corridors of width typical for large buildings, significant levels of nonadherence to social distancing guidance will inevitably occur, especially for the “six-feet rule” (compare, e.g., against the work on exposure time in Ref. 36). Given the clear value of realistic and actionable public-health guidance (both in terms of improving rates of adherence and in terms of reducing social unease for individuals attempting to adhere to official guidance), these results can play a helpful role in informing practical public-health messaging on social distancing as shared spaces return to pre-pandemic levels of pedestrian density.

## IV. CONCLUSIONS AND OUTLOOK

In this work, we have studied the effect of social distancing (as captured by an attenuation length scale for an exponentially decaying interaction potential within a social-force model) on the steady dynamics of a pedestrian counterflow within corridors of various fixed widths. By running high-throughput particle-based simulations, we have constructed fundamental diagrams (with quantified uncertainties) that illustrate how average pedestrian speed falls with increasing density and increasing degree of social distancing. We find that as pedestrian density and degree of social distancing increase, pedestrian flows undergo significant changes in their steady dynamics that are qualitatively and quantitatively reminiscent of a vapor-to-liquid-to-solid phase transition. Our primary takeaway is that pedestrian congestion is a likely outcome of strict adherence to (even modest) increases in social distancing.

This work suggests several natural avenues for further inquiry. For example, given many anecdotal and empirical reports of uneven levels of compliance with social distancing guidelines (see, e.g., Ref. 37) one might wonder: What is the impact of heterogeneous preferences for social distancing on the steady dynamics of a pedestrian flow? In the granular media community, it is very well known that jamming and crystallization phenomena in polydisperse disks can differ significantly as compared to monodisperse disks (see, e.g., Ref. 38). It is possible that qualitatively similar trends can be observed in pedestrian flows. More broadly speaking, it is worthwhile to study the role of heterogeneous preferences in pedestrian flows (see, e.g., recent work investigating variations in pedestrian target speed^39^).

Although the length scales in this work are motivated specifically by public-health guidance associated with the COVID-19 pandemic, these results are broadly applicable to any situation in which a pedestrian population undergoes a change in preference for social distancing (due, e.g., to concerns about SARS-CoV-2 variants, other diseases, or for cultural or religious reasons). Given that societal awareness of social distancing as a strategy for reducing disease transmission is higher today than before the pandemic, it is possible that some individuals will exhibit seasonal variations in *d*_0_ (with increases aligning with, e.g., the start of flu season).

It is also worth remarking that the central thrusts of this study bear more than a passing resemblance to the animating ideas behind work in the fields of nano- and micro-fluidics; in particular, both fields place a large emphasis on novel transport phenomena (see, e.g., Refs. 40 and 41) that emerge as a consequence of a characteristic internal length scale of the flowing medium (e.g., the van der Waals radius of a fluid molecule, the hydrodynamic radius of a polymer, or the social distance maintained by a pedestrian) approaching the characteristic length scale of the confining environment (e.g., the width of a nano-/micro-channel or a hallway). As such, we believe that it is likely that researchers working in the fields of confined flows and particulate flows can contribute meaningful physical insights to the field of pedestrian dynamics and, more broadly, to the dynamics and statistical physics of urban systems (see, e.g., Refs. 42 and 43).

Finally, experimental data characterizing the effect of social distancing on confined pedestrian flows would (needless to say) be logistically challenging and ethically dubious to obtain amidst the ongoing pandemic. However, as shared spaces (e.g., school campuses, offices, entertainment venues) return to higher densities in late 2021 through 2022, it will be useful to assess the degree to which pedestrians continue exhibiting a preference for social distancing length scales greater than pre-pandemic values. To this end, field studies and parameter-inference approaches^44,45^ will be especially valuable.

## Data Availability

The data that support the findings of this study are available from the corresponding author upon reasonable request.

## APPENDIX: PEDESTRIAN DYNAMICS SIMULATIONS

In this Appendix, we provide details on the specific numerical implementation of our simulations. We first address the last two terms in Eq. (1). In particular, the near-field force on pedestrian *i* due to pedestrian *j* is given, as a function of their center-of-mass separation *r_ij_*, by

$${\vec{F}}_{\text{near}}(r_{ij})=-k_{\text{normal}}{\tilde{r}}_{ij}\theta ({\tilde{r}}_{ij}){\hat{n}}_{ij}-\mu_{\text{tangential}}{\tilde{r}}_{ij}\theta ({\tilde{r}}_{ij})\Delta v_{ij,\text{tangential}\;}{\hat{t}}_{ij},$$

where ${\tilde{r}}_{ij}\equiv 2r_{0}-r_{ij},\;2r_{0}$ is a characteristic “diameter” for a pedestrian (we use $2r_{0}=0.3$ m throughout), $k_{\text{normal}}$ and $\mu_{\text{tangential}}$ are prefactors that set the scales of the normal and tangential forces (we use $k_{\text{normal}}=1.2\times 10^{5}$ N/m and $\mu_{\text{tangential}}=2.4\times 10^{5}$ Pa s throughout), *θ* is the Heaviside step function, ${\hat{n}}_{ij}$ is the unit vector pointing from *i* to *j*, ${\hat{t}}_{ij}$ is the unit normal to ${\hat{n}}_{ij}$, and $\Delta v_{ij,\text{tangential}}\equiv ({\vec{v}}_{i}-{\vec{v}}_{j})\cdot {\hat{t}}_{ij}$ is the projection of the velocity difference between *i* and *j* onto the unit normal. We note that the specific numerical values for the prefactors are based upon Helbing *et al.*^25^ The random contribution $\vec{R}$ is modeled as a stationary Gaussian process with mean of zero, variance of 2 square-Newtons, and no correlation in time. This process is implemented in our LAMMPS simulations using a Langevin thermostat,^46,47^ which supplies two additional forces to each pedestrian: (1) a fluctuating force with magnitude $\sqrt{\frac{2}{dt}\cdot k_{B}T\cdot \frac{m}{\gamma }}N(0,1)$ (and uniformly random orientation) and (2) a dissipative force with magnitude $\frac{m}{\gamma }||{\vec{v}}_{\text{inst}}||$ (oriented opposite to ${\vec{v}}_{\text{inst}}$). Here, $k_{B}T$ is the thermal energy corresponding to the thermostat temperature (we use $k_{B}T=0.6J$); *γ* is a timescale for dissipation (we use $m/\gamma =5\times 10^{-3}$ kg/s); N(0,1) is a Gaussian random variable with zero mean and unit variance; and, as a reminder, *dt* is the time step (we use $dt=3\times 10^{-3}$ s). We note that the dissipative force is of negligible magnitude in our simulations since maximum pedestrian speeds are O(1) m/s, and so these forces are approximately three orders of magnitude smaller in magnitude than typical magnitudes of the fluctuating force.

Each simulation is initialized by placing pedestrians on a hexagonal lattice (with a lattice constant of 0.9 m) that spans 30 m of the 40 m of total hallway length. Pedestrians walking in each direction are initially segregated from each other, with an initial separation distance of 10 m. From this initial lattice, an equal number of pedestrians are removed at random from both the right-walking and left-walking populations, until the target value of $\phi_{\text{exc}}$ is achieved. Initial velocities are set as zero for all pedestrians.

Simulations were performed on an 80-core server consisting of Intel Xeon Gold processors.
